# Identification of genes and gene pathways associated with major depressive disorder by integrative brain analysis of rat and human prefrontal cortex transcriptomes

**DOI:** 10.1038/tp.2015.15

**Published:** 2015-03-03

**Authors:** K Malki, O Pain, M G Tosto, E Du Rietz, L Carboni, L C Schalkwyk

**Affiliations:** 1 MRC Social, Genetic and Developmental Psychiatry Centre, Institute of Psychiatry, King's College London, London, UK; 2 Laboratory for Cognitive Investigations and Behavioural Genetics, Tomsk State University, Tomsk, Russia; 3 Department of Pharmacy and Biotechnology, Alma Mater Studiorum, University of Bologna, Bologna, Italy; 4 School of Biological Sciences, University of Essex, Colchester, UK

## Abstract

Despite moderate heritability estimates, progress in uncovering the molecular substrate underpinning major depressive disorder (MDD) has been slow. In this study, we used prefrontal cortex (PFC) gene expression from a genetic rat model of MDD to inform probe set prioritization in PFC in a human post-mortem study to uncover genes and gene pathways associated with MDD. Gene expression differences between Flinders sensitive (FSL) and Flinders resistant (FRL) rat lines were statistically evaluated using the RankProd, non-parametric algorithm. Top ranking probe sets in the rat study were subsequently used to prioritize orthologous selection in a human PFC in a case–control post-mortem study on MDD from the Stanley Brain Consortium. Candidate genes in the human post-mortem study were then tested against a matched control sample using the RankProd method. A total of 1767 probe sets were differentially expressed in the PFC between FSL and FRL rat lines at (*q*⩽0.001). A total of 898 orthologous probe sets was found on Affymetrix's HG-U95A chip used in the human study. Correcting for the number of multiple, non-independent tests, 20 probe sets were found to be significantly dysregulated between human cases and controls at *q*⩽0.05. These probe sets tagged the expression profile of 18 human genes (11 upregulated and seven downregulated). Using an integrative rat–human study, a number of convergent genes that may have a role in pathogenesis of MDD were uncovered. Eighty percent of these genes were functionally associated with a key stress response signalling cascade, involving *NF-κB* (nuclear factor kappa-light-chain-enhancer of activated B cells), *AP-1* (activator protein 1) and *ERK/MAPK,* which has been systematically associated with MDD, neuroplasticity and neurogenesis.

## Introduction

Major depressive disorder (MDD) is a severe psychiatric disease providing a significant contribution to the global burden of disease.^1, 2^ Endeavours to identify factors underlying the molecular basis of MDD have been guided by quantitative studies reporting a substantial genetic contribution to its development.^3^ However, as with other Axis-I psychiatric disorders, progress in identifying the genetic variation associated with the pathology have been slow.^4, 5^

One approach for studying the molecular mechanism of MDD in disease-relevant brain regions is by exploring messenger RNA (mRNA) changes in animal models.^6^ The identification of differentially expressed genes can provide early clues into the molecular mechanisms associated with the pathology in humans. However, the exploration of the human brain transcriptome has major limitations, namely the sample limitations, including the requirement of post-mortem brain tissue and confounding factors.^7^ Indeed, several studies have demonstrated the advantage of using animal models of disease to inform human studies by providing a hypothesis-free candidate genes selection with higher prior probability of being involved in the human pathology.^6, 8, 9^

In this study, we explored expression differences between Flinders Sensitive or Resistant Lines (FSL/FRL) of rat, which represent one of the most robust genetic models of MDD.^10^ Flinders rat have been selectively bred to display a high sensitivity to diisopropyl fluorophosphates and cholinergic agents, mimicking an established neurobiological feature of MDD in humans.^11^ In addition, these lines have been reported to exhibit a number of other characteristic biological and behavioural features of MDD.^10, 12^

By identifying differentially expressed genes between FSL and FRL lines, it is possible to guide candidate gene selection for subsequent analysis in human post-mortem samples.^8^ A recently published study from the Genome-based Therapeutic Drugs for Depression (GENDEP) consortium previously explored hippocampal expression differences by adopting this approach.^8^ However prefrontal cortex (PFC) expression profiles are now available and existing evidence demonstrates that abnormalities have been repeatedly reported in MDD patients in this brain region.^13, 14^ Therefore, we followed a similar design to investigate gene expression changes in PFC. In this study, identification of differentially expressed genes in the PFC of FSL/FRL was used to inform probe set selection in a comparable human PFC mRNA data set. We hypothesized that a set of genes differentially expressed in the genetic rat MDD model would also be differentially regulated in a human, case–control study on MDD.

## Materials and methods

### Design

This study used transcriptomic data derived from the PFC of a rat model of MDD, investigated within the GENDEP project (http://gendep.iop.kcl.ac.uk), to guide candidate gene selection for subsequent analysis within a comparable post-mortem case–control study on MDD from the Stanley Brain Consortium (http://www.stanleyresearch.org). GENDEP is a multicentre pharmacogenetic project consisting of a series of studies involving humans, animal models and *in vitro* experiments. GENDEP design was aimed at performing an integrative analysis of key processes to provide insight into the molecular mechanisms underlying MDD and the differential response to antidepressant treatment. One subgroup of rodent studies within the GENDEP project involved comparison of mRNA levels in the PFC of FSL and FRL rat lines, a robust model of ‘endogenous' depression. Candidate modulated genes identified in the animal model were subsequently validated in human samples. The case–control study of MDD by the Stanley Brain Consortium similarly collected information of key molecular processes within post-mortem tissue derived from participants using exclusion criteria which can be found on the Stanley Brain Consortium website (http://www.stanleyresearch.org).

### Animals

This study used 39 adult rats consisting of two strains, 17 FSL and 22 FRL.^15^ Rats were bred in Stockholm at the Karolinska Institutet. Animal maintenance and experimental procedures were conducted in accordance to the European Communities Council Directive of 24 November 1986.

### Human samples

This study used human samples, which have been made available to researchers worldwide, after being donated to the Stanley Foundation Brain Collection in MD, USA. The data were downloaded from the Gene Expression Omnibus (accession ID: GSE12654; www.ncbi.nlm.nih.gov/geo). Dissection of PFC tissues (Brodmann's Area 10) and microarray procedures were carried out by Iwamoto *et al.*^16^ Post-mortem PFCs from individuals diagnosed with MDD were carefully matched with controls. Diagnoses of MDD were in accord with the Diagnostic and Statistical Manual of Mental Disorders, Fourth Edition.^17^ Controls were matched by age, gender, race, pH, post-mortem interval and quality of mRNA extraction. Consistent with previous studies using this data set,^16^ samples were excluded if mRNA quality was poor and individuals were >65 years of age leading to a total of 26 human PFC samples (11 cases and 15 controls) being used. Further information on the human data and original microarray procedure can be found elsewhere.^16^

### mRNA extraction and lab protocols

This study used raw gene expression (.CEL) files from data collected previously. PFC mRNA protocols extraction from Flinders rats has been described elswhere.^15^ cRNA was hybridized to Affymetrix Rat Genome 230 2.0 arrays (Santa Clara, CA, USA). The Affymetrix Rat Genome 230 2.0 GeneChip provides a comprehensive coverage of the rat genome containing 31,000 probe sets representing 28,000 genes.

Information on The Stanley Foundation human brain collection and Neuropathology Consortium can be found elsewhere.^18^ PFC (Brodmann's Area 10) tissue using the Trizol method and then purified using the Qiagen RNeasy kit (Hilden, Germany). RNA (8–10 mg) was used to synthesize cDNA, which was then used to generate cRNA. PFC (Brodmann's Area 10) cRNA was hybridized onto the Affymetrix HG-U95A. A detailed description of the microarray procedure has been previously described by Iwamoto *et al.*^16^

### Statistical analysis procedure

Rat data set: Raw.CEL files for FSL and FRL rats were downloaded from the Gene Expression Omnibus (GEO; accession number GS2088; www.ncbi.nlm.nih.gov/geo). Initially raw data was normalized and summarized using RMA (Robust Multichip Averaging), returning log2 transformed intensities.^19^ The rat data were collected in two cohorts requiring ComBat merging function within the inSilicoMerging package to avoid potential batch effects.^20^ To identify top differentially expressed probe sets within the summarized data, the PRadvance function within the RankProd package was used.^21^ *P*-values of differential expression were determined using 10 000 permutations. To select a list of candidate genes to carry forward for human analysis, a conservative false discovery rate (pfp, percentage false positive) threshold of *P*<0.001 and a fold change >1.5 was used. To match probe sets between rat and human data sets, gene symbols were used. A list of gene symbols corresponding to significant rat probe sets was determined using PANTHER (http://www.pantherdb.org).

Human data set: The raw.CEL files for the control and MDD diagnosed human post-mortem samples were downloaded from the Gene Expression Omnibus (accession ID: GSE12654; www.ncbi.nlm.nih.gov/geo). Initially raw data were normalized and summarized using RMA, returning log_2_ transformed intensities.^19^ Human genes orthologous to the candidate genes selected by prior rat data set analysis were determined using the NetAffx Rat 230 v2 orthologue annotation. Top differentially expressed genes between the control and MDD individuals determined using the PRadvance function within the RankProd package was used.^21^ A significance threshold of pfp <0.05 was calculated using 10 000 permutations. Fold change in expression was calculated manually for MDD-associated probes.

Probe sets differentially expressed in the human sample were subsequently uploaded to the Ingenuity Pathway Analysis (IPA) software. The IPA software was used for its ability to analyse mRNA data in the context of known biological response and regulatory networks. IPA returns a score for each network using a *P*-value based on Fisher's exact test. The software determines the probability that each biological function assigned to that data set was due to chance alone.

## Results

First, we statistically evaluated mRNA differences between Flinders Sensitive and Resistant rats. The RankProd non-parametric algorithm uncovered 1767 probe sets on the Affymetrix Rat Genome 230 2.0 Gene Chip, differentially regulated at the stringent significance threshold of pfp ⩽0.001, and a fold change ⩾1.5. Given the potential for false positives, a conservative threshold was preferred to prioritize probe sets with higher prior probability of association with MDD in the human study. The results from the rat study were used to provide a list of candidate probe sets for RankProd analysis in the human data set. A total of 898 probes sets, tagging the expression profile of the genes orthologous to ones differentially expressed in rat, were also found on Affymetrix's HG-U95A chip using the NetAffx tool from Affymetrix.

We then obtained a subset of the human mRNA data set to include only those probe sets prioritized from the animal study. To statistically evaluate differences between post-mortem MDD cases and controls, we used the PRadvance function in the RankProd function using the single origin option. RankProd analysis of human data set uncovered 18 probe sets differentially expressed in the PFC between humans diagnosed with MDD and control at the corrected significant threshold of (pfp ⩽0.05, *P*-value ⩽6 × 10^−^^4^, Table 1). A graph showing the distribution of pfp values for up- and downregulated probe sets can be found in Supplementary Figure 1.

The analysis uncovered a total of 15 genes, 11 significantly upregulated and four significantly downregulated in MDD cases versus control. The list includes several genes previously implicated in depression including the *NTRK2, AXL* and *TAC1* genes. Affymetrix's arrays are designed to have multiple probe sets tagging the expression of different genes in the 3'-UTR region. Different probe sets tagging the expression of the same genes were among the top ranking probe sets further reducing the chance of false positives. The fold change of significant probes ranged from 1.18 to 1.80. As the findings are in human brain, moderate fold changes in gene expression are expected due to large heterogeneity and the relatively sensitive neural environment. Last, we uploaded all 20 probes sets on the IPA software. Nineteen probe sets were mapped to the ingenuity reference database and carried forward for analysis. The top ranking pathway returned by IPA, with a score of 26 and included 12 out of 15 reference molecules uploaded. The majority of the genes uncovered (80%) are significantly associated with same network centred on the NF-κb (nuclear factor kappa-light-chain-enhancer of activated B cells), MapK and ERK signalling cascade. This neurogenetic-neurotrophic pathway is of particular interest and relevance as it has been extensively associated with MDD and stress response.

## Discussion

This study has used transcriptomic data derived from the PFC of FSL and FRL rat strains to guide candidate gene selection in human samples generated in the corresponding brain region and subsequently identify molecular dysregulations associated with MDD in the PFC of humans. Analysis of differential gene expression within the PFC of the selectively bred FSL and FRL strains, a robust model of MDD, provided an appropriate number of candidate genes to be then validated in the more relevant human data set. The analysis of differential gene expression in the PFC of individuals diagnosed with MDD or controls, using the candidate gene selection, provided a list of genes and biological pathways associated with MDD.

**Figure 1:**
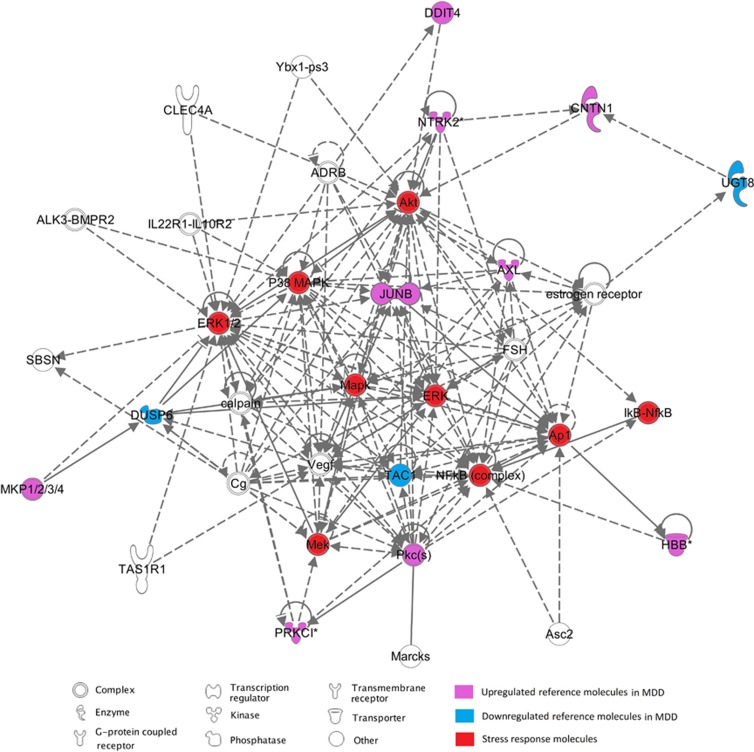
Network uncovered using IPA. The network includes 12 out of the 15 genes uploaded on the Ingenuity Pathway Analysis reference database. These genes are functionally related and have a role in the same network centred on NF-κb, MAPK and ERK signalling cascade. This network has previously been implicated in both MDD studies and in studies on response to antidepressant treatment. Genes central to the stress response signalling cascade have been highlighted in red. Genes identified as differentially expressed in MDD individuals have been highlighted: pink indicated upregulation and blue indicates downregulation. IPA, Ingenuity Pathway Analysis; MDD, major depressive disorder; NF-κb, nuclear factor kappa-light-chain-enhancer of activated B cells.

IPA identified a functional pathway involving 80% of the genes significantly associated with MDD in humans (Figure 1). This pathway was centred on key stress response signalling molecules, such as *NF-κB*, *AP-1* (activator protein 1) and several *MAPKs* (mitogen activated protein kinases). These *MAPKs* function to cascade signals, in response to extracellular stimuli, regulating the activity of a number of transcription factors including *AP-1* and *NF-κB* (Figure 1). Taking *NF-κB* as an example, upon activation, the transcription factor translocates into the nucleus where it binds to target DNA sequences (kB sites). *NF-κB* binding to these motifs results in the transcriptional regulation of a large number of pro-inflammatory genes encoding proteins such as cytokines.^22^ As a result of such processes, a range of other downstream pathways are modulated to adapt cellular/tissue function with regard to the transduced signals. In particular, pathway analysis identified downstream neuroplasticity and neurogenesis pathways as dysregulated in the PFC of MDD individuals. These neurogenetic pathways involved a number of genes identified by our RankProd analysis (for example, *NTRK2, AXL* and *CNTN1*) as well as molecules based upon functional enrichment (for example, vascular endothelial growth factor). Supporting the validity of this enrichment, vascular endothelial growth factor has been previously implicated in hippocampal neurogenesis and response to stress.^23, 24^ This powerful approach for the enrichment of probe sets has been able to encapsulate and support a molecular model of depression involving inflammation-mediated dysregulation of neuroplasticity and neurogenesis. Further support of this interpretation of the findings is apparent when the differentially regulated genes returned by RankProd analysis are functionally annotated separately.

A number of genes differentially regulated in MDD individuals indicate increased activation of stress and oxidative response pathways in the PFC. Two probes within *PKCI* (protein kinase C Iota) were identified as upregulated in MDD. *PKCI* encodes a serine/threonine protein kinase, which has been reported to regulate *NF-κB* activity and neurotrophin-mediated neuronal differentiation and survival via neuronal growth factor.^25^ *PKCI* has been previously associated with MDD in a previous analysis using the same human data set.^26^ *PKCI* has also been determined as upregulated in suicidal individuals, compared with non-suicidal individuals, both with mood disorders.^27^ These findings support PKCI as a modulator of mood disorders.

Another gene associated with MDD involved in the stress response is *DDIT4. DDIT4* (DNA-damage-inducible transcript 4), also known as *REDD1*, is an inhibitor of *mTORC1* (mammalian target of rapamycin complex-1) and it is regulated by oxidative stress. Recent findings demonstrated *DDIT4* involvement:^28^ *DDIT4* was activated in the PFC of rats in response to stress, viral-mediated upregulation of *DDIT4* in the PFC of rats induced depressive-like and anxiety-like behaviours, and the analysis of different post-mortem MDD samples showed an upregulation of *DDIT4* in the PFC of MDD individuals. Our findings are in agreement with the upregulation of *DDIT4* in the PFC of MDD patients, highlighting the potential importance of *mTORC1* pathways in MDD.

One of the main theories of the pathophysiology of MDD asserts that the exposure to chronic stress alters transcriptional regulation of growth factors and hormones leading to impaired neurogenesis and neuroplasticity.^29^ According to this theory, following our findings of altered stress response pathways, genes involved in neuroplasticity and neurogenesis were also expected to be differentially expressed. Compelling evidence showed an association between dysregulation of neurotrophic pathways, impaired neuronal plasticity and MDD.^30, 31^ The neuroplastic functions of the neurotrophic pathway are centred on brain-derived neurotrophic factor (BDNF), its receptor neurotrophic tyrosine receptor kinase type 2 (*NTRK2* or *TrkB*) and the transcription factor *cAMP* (cyclic adenosine monophosphate) binding protein 1 (*CREB1*) which regulates both the expression of *BDNF* and the *TrkB* gene.^32^ In this study, two key genes in the neurotrophic pathway were associated with MDD in the PFC, in agreement with this theory. In our results, the clearest evidence for the dysregulation of neurotrophic pathways in MDD comes from the finding of an upregulation of the NTRK2 gene in MDD patients as shown by two probes. The *NTRK2* gne encodes the neurotrophic tyrosine receptor kinase type 2 (*TrkB*), which binds BDNF as well as other neurotrophins, including NT-4 (neurotrophin-4) and NT-3 (neurotrophin-3). Previous studies have reported that antidepressant treatment leads to an upregulation of BDNF expression and activation of *NTRK2* via *CREB1.*^33, 34^ Moreover, genetic variation within and downregulation of *NTRK2* in the PFC has been associated with suicidal ideation and suicide,^35, 36^ supporting the role of *NTRK2* in depressive symptoms.

Another gene significantly upregulated in MDD patients was *PDE4DIP* (phosphodiesterase 4D interacting protein). *PDE4DIP*'s function is to compartmentalize *PDE4D* within the *cAMP* pathway.^37^ *PDE4* proteins degrade *cAMP* and are important for the regulation of intracellular *cAMP* concentrations. Antidepressant effects exerted by *PDE4* inhibitors through the enhancement of *cAMP* signalling are mainly attributable to the inhibition of *PDE4D.*^38^ It is conceivable that our finding of *PDE4DIP* upregulation in MDD individuals indicates increased compartmentalization of *PDE4D* leading to altered *cAMP* availability. This provides a link between *PDE4DIP* and neurotrophin regulation via *CREB1*.

The *AXL* gene, encoding *AXL* receptor protein-tyrosine kinase (*RPTK*) was shown in our analysis to be upregulated in MDD individuals. This subfamily of *RPTK*s has been less well characterized in the brain than the previously discussed neurotrophic receptor tyrosine kinases. The *AXL RPTK*s acts as the receptor for *Gas6* (growth arrest-specific gene-6), which is expressed throughout the adult central nervous system. A study investigating the function of *AXL RTPK*s in the adult rat brain suggested a role in neuronal survival and growth, and regulating synaptic function and plasticity.^39^ Further research is required to determine the extent to which these *RPTK*s regulate the molecular mechanisms of depression, however, the *AXL* gene identified as a biomarker for depressive symptoms in elderly individuals.^40^

TAC1 (tachykinin, precursor 1), reported as downregulated in MDD individuals, is a complex gene encoding four proteins within the tachykinin hormone family. These four proteins, called substance P, neurokinin A, neuropeptide K and neuropeptide gamma, function as neurotransmitters and neuromodulators. Our finding is supported by a study reporting that *Tac1* knockout in mice led to reduced anxiety-like and depression-like behaviours.^41^ In addition, antagonists of the tachykinin receptor, called *TACR1*, have been investigated in clinical trials for depression with mixed results.^42^ Research of *TACR1* antagonists is still under investigation for antidepressant potential. Further research into the protein products of *TAC1* and other neurokinins could provide insight into the molecular mechanisms of MDD.

*DUSP6* (dual-specificity protein phosphatase 6) was identified in this study, as downregulated in the PFC of MDD individuals. The downregulation of *DUSP6* has also been reported in the PFC of individuals with bipolar disorder.^43^ Furthermore, genetic variation within and proximal to *DUSP6* has been associated with bipolar disorder and lithium-induced *ERK* activation,^44^ a mechanism related to neuroplasticity and neurogenesis,^45^ suggesting a role for *DUSP6* in the regulation of mood disorders, neuroplasticity and neurogenesis.

Another pathway implicated in depression with effects on neuroplasticity and neurogenesis is under the regulation of the growth factor erythropoietin, both independent and dependent on its role in haematopoiesis.^46, 47^ Our results provide some evidence that erythropoietic pathways are altered in MDD individuals with the three most significantly upregulated probe sets representing two genes, *HBA* and *HBB* (haemoglobin alpha and beta). This finding is quite interesting as it could be interpreted in a number of ways. It is possible that the upregulation of these haemoglobin genes is indicative of an increased concentration of haemoglobin in the PFC of individuals with MDD. However, another more likely interpretation of these findings includes a negative feedback loop where decreased haemoglobin in the PFC of individuals with MDD initiates an upregulation of haemoglobin genes. For example hypoxia induces an upregulation of *HIF1* (hypoxia inducible factor) and subsequently *HBA* and *HBB.*^48, 49^

Low levels of systemic haemoglobin have symptoms of high levels of fatigue and lethargy, similar to symptoms seen in some depressed individuals. Low peripheral haemoglobin levels in cancer patients have been reported to correlate with severity of depression,^50^ even when controlling for severity of cancer.^51^ Moreover, it was reported that a low level of haemoglobin was a significant risk factor for post-partum depression even when controlling for confounding variables such as delivery complications and perinatal blood loss.^52^ However, measures of haemoglobin in the brain would be required to establish a connection with depression. One study used near-infrared spectroscopy to measure concentrations of oxygenated haemoglobin, as a parameter for activity, in the PFC of MDD individuals and controls.^53^ This study reported a significant decrease in oxygenated haemoglobin in the PFC of MDD individuals, particularly in Brodmann's area 10. This finding and our result of upregulated haemoglobin genes in Brodmann's area 10 suggest low levels of anaemia in the PFC of depressed individuals requiring further research. It is possible that if this anaemia does occur in the brain, it could initiate stress response pathways via hypoxia-induced factors.^54^

### Strengths and limitations

The first limitation to this study is its use of post-mortem tissue for the isolation of human PFC RNA. RNA rapidly degrades and therefore isolation must be carried out carefully to preserve the quality of the sample. In addition, due to the environmental sensitivity of gene expression, the process of death could cause global transcriptomic changes throughout the body creating artefacts in the results. However, without the use of post-mortem tissue, it is not possible to extract RNA from MDD-relevant brain tissue in humans. We have applied an integrative cross-species approach, which helps to overcome these issues by selecting candidate genes from the analysis of the FSL model in which relevant brain tissue is accessible and environmental factors can be controlled. Although relatively stringent thresholds were used throughout the analysis, further replication of findings may be required.

Second, the use of an animal model to study complex behavioural phenotypes in humans is limited, due to the impossibility to reproduce all the complex features of a psychiatric disease in rodents. However animal models have been shown to be an important source of information for the study of depression and antidepressant treatment response in humans.^9^

Third, although we used a cross-validation method by exploring mRNA differences across different studies, verification of findings with other experimental methods such as quantitative PCR would have been desirable; results should be interpreted in light of this limitation.

Last, the human data were derived from just one subsection of the PFC; Brodmann's area 10, meaning this study was unable to see how gene expression changes associated with MDD varied in other subsections of the PFC.

In summary, the findings highlight the importance of stress response pathways leading to altered regulation of neurogenetic and neurotrophic pathways such as the neurotrophin and neurokinin pathways, supporting previous reports of increased stress response and impaired neuroplasticity underlying the pathophysiology of MDD. Further research on the mechanism by which these molecules alter depressive behaviour could improve prevention, diagnosis and treatment of MDD.

## Figures and Tables

**Table 1:** Table shows probe ID, gene title, gene symbol, FC (fold change), pfp (percentage of false positives) and *P*-value for probes significantly associated with MDD

*Probe ID*	*Gene title*	*Gene symbol*	*FC*	*pfp*	P*-value*
*Upregulated probe sets*					
31525_s_at	Haemoglobin, alpha 1 /// haemoglobin, alpha 2	HBA1 /// HBA2	1.80	0.0E+00	<1.00E−06
32052_at	Haemoglobin, beta	HBB	1.67	0.0E+00	<1.00E−06
31687_f_at	Haemoglobin, beta	HBB	1.65	0.0E+00	<1.00E−06
40928_at	WD repeat and SOCS box containing 1	WSB1	1.35	9.2E−03	<1.00E−06
38280_s_at	Neurotrophic tyrosine kinase, receptor, type 2	NTRK2	1.28	1.1E−02	1.00E−04
1603_g_at	Protein kinase C, iota	PRKCI	1.35	1.7E−02	1.00E−04
33182_at	Neurotrophic tyrosine kinase, receptor, type 2	NTRK2	1.27	2.5E−02	2.00E−04
31809_at	Contactin 1	CNTN1	1.22	2.3E−02	2.00E−04
36059_at	Low density lipoprotein receptor-related protein 4	LRP4	1.31	3.1E−02	3.00E−04
1602_at	Protein kinase C, iota	PRKCI	1.32	3.0E−02	3.00E−04
33364_at	Phosphodiesterase 4D interacting protein	PDE4DIP	1.22	2.7E−02	3.00E−04
32786_at	jun B proto-oncogene	JUNB	1.33	2.6E−02	4.00E−04
1278_at	AXL receptor tyrosine kinase	AXL	1.28	3.8E−02	6.00E−04
39827_at	DNA-damage-inducible transcript 4	DDIT4	1.27	3.8E−02	6.00E−04
					
*Downregulated probe sets*					
36363_at	UDP glycosyltransferase 8	UGT8	1.22	2.0E−04	<1.00E−06
36490_s_at	Phosphoribosyl pyrophosphate synthetase 1	PRPS1	1.28	2.2E−02	<1.00E−06
41193_at	Dual specificity phosphatase 6	DUSP6	1.18	2.0E−02	1.00E−04
36254_at	Tachykinin, precursor 1	TAC1	1.27	3.2E−02	1.00E−04

Abbreviation: MDD, major depressive disorder.
