# Supplementary figures and images for: Identification of genes and gene pathways associated with major depressive disorder by integrative brain analysis of rat and human prefrontal cortex transcriptomes
Source: Transl Psychiatry. 2015 Mar 3;5(3):e519–. doi: 10.1038/tp.2015.15 (PMC4429169; doi:10.1038/tp.2015.15)

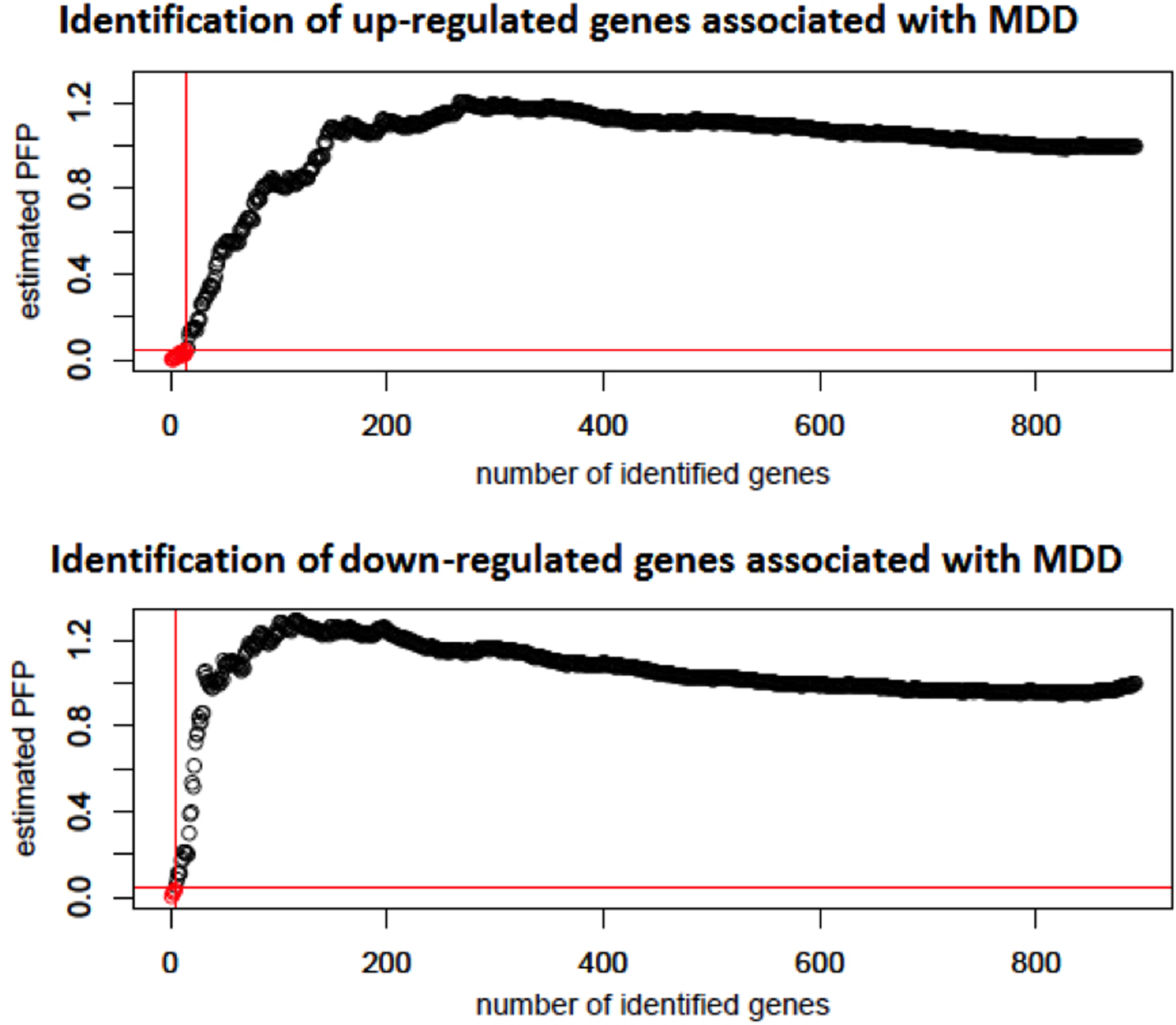

Supplement: Supplementary Figure S1 [file tp201515x1.tif]
